# Association of an Emergency Department–Based Intensive Care Unit With Survival and Inpatient Intensive Care Unit Admissions

**DOI:** 10.1001/jamanetworkopen.2019.7584

**Published:** 2019-07-24

**Authors:** Kyle J. Gunnerson, Benjamin S. Bassin, Renee A. Havey, Nathan L. Haas, Cemal B. Sozener, Richard P. Medlin, Jennifer A. Gegenheimer-Holmes, Stephanie L. Laurinec, Caryn Boyd, James A. Cranford, Sage P. Whitmore, Cindy H. Hsu, Reham Khan, Neha N. Vazirani, Stephen G. Maxwell, Robert W. Neumar

**Affiliations:** 1 Department of Anesthesiology, Michigan Medicine, University of Michigan, Ann Arbor; 2 Department of Emergency Medicine, Michigan Medicine, University of Michigan, Ann Arbor; 3 Division of Emergency Critical Care, Michigan Medicine, University of Michigan, Ann Arbor; 4 Michigan Center for Integrative Research in Critical Care, Ann Arbor; 5 Department of Internal Medicine, Michigan Medicine, University of Michigan, Ann Arbor; 6 Department of Surgery, Michigan Medicine, University of Michigan, Ann Arbor; 7 School of Dentistry, University of Michigan, Ann Arbor

## Abstract

**Importance:**

Increased patient acuity, decreased intensive care unit (ICU) bed availability, and a shortage of intensivist physicians have led to strained ICU capacity. The resulting increase in emergency department (ED) boarding time for patients requiring ICU-level care has been associated with worse outcomes.

**Objective:**

To determine the association of a novel ED-based ICU, the Emergency Critical Care Center (EC3), with 30-day mortality and inpatient ICU admission.

**Design, Setting, and Participants:**

This retrospective cohort study used electronic health records of all ED visits between September 1, 2012, and July 31, 2017, with a documented clinician encounter at a large academic medical center in the United States with approximately 75 000 adult ED visits per year. The pre-EC3 cohort included ED patients from September 2, 2012, to February 15, 2015, when the EC3 opened, and the post-EC3 cohort included ED patients from February 16, 2015, to July 31, 2017. Data analyses were conducted from March 2, 2018, to May 28, 2019.

**Exposures:**

Implementation of EC3, an ED-based ICU designed to provide rapid initiation of ICU-level care in the ED setting and seamless transition to inpatient ICUs.

**Main Outcomes and Measures:**

The main outcomes were 30-day mortality among ED patients and rate of ED to ICU admission.

**Results:**

A total of 349 310 visits from a consecutive sample of ED patients (mean [SD] age, 48.5 [19.7] years; 189 709 [54.3%] women) were examined; the pre-EC3 cohort included 168 877 visits and the post-EC3 cohort included 180 433 visits. Implementation of EC3 was associated with a statistically significant reduction in risk-adjusted 30-day mortality among all ED patients (pre-EC3, 2.13%; post-EC3, 1.83%; adjusted odds ratio, 0.85; 95% CI, 0.80-0.90; number needed to treat, 333 patient encounters; 95% CI, 256-476). The risk-adjusted rate of ED admission to ICU decreased with implementation of EC3 (pre-EC3, 3.2%; post-EC3, 2.7%; adjusted odds ratio, 0.80; 95% CI, 0.76-0.83; number needed to treat, 179 patient encounters; 95% CI, 149-217).

**Conclusions and Relevance:**

Implementation of a novel ED-based ICU was associated with improved 30-day survival and reduced inpatient ICU admission. Additional research is warranted to further explore the value of this novel care delivery model in various health care systems.

## Key Points

**Question:**

How is implementation of an emergency department (ED)–based intensive care unit (ICU) associated with mortality among ED patients and with inpatient ICU admission?

**Findings:**

In this cohort study of 349 310 patient encounters in the ED of an academic medical center, implementation of an ED-based ICU was associated with reductions in risk-adjusted 30-day mortality among ED patients, from 2.13% to 1.83%, and ICU admissions, from 3.2% to 2.7% of all ED visits.

**Meaning:**

Implementation of an ED-based ICU was associated with improved survival and reduced inpatient ICU admissions.

## Teaser

This cohort study compares 30-day mortality and inpatient intensive care unit (ICU) admissions before and after the implementation of a novel emergency department–based ICU.

## Introduction

Critical illnesses and injuries account for 40% of total annual US hospital costs and $260 billion in annual cost to the US economy.^1^ Increased patient acuity, decreased intensive care unit (ICU) bed availability, and a persistent shortage of intensivist physicians have strained ICU capacity in many health care systems.^2^ This combination of factors has created a greater demand to deliver prolonged critical care in the emergency department (ED) setting. From 2001 to 2009, the total annual hours of critical care delivered in US EDs increased from 3.2 million to 10.1 million hours, and ICU admissions from the ED nearly doubled, from 1.2 million to 2.2 million per year.^3^

Multiple studies have demonstrated that increased ED boarding time is associated with worse outcomes for patients requiring ICU-level care.^4,5,6,7,8,9,10^ Patients who are critically ill and experience delay longer than 6 hours from arriving at the ED to ICU transfer have increased hospital length of stay (LOS) and higher ICU and hospital mortality.^4^ In the United States, 33% of ICU admissions from the ED have an ED LOS longer than 6 hours.^3^

In response to this challenge, health care systems are beginning to develop and implement novel strategies, including ED-based ICUs, to optimize critical care delivery outside of the traditional inpatient ICU structure.^11,12^ Because these strategies require substantial resources, it is critical to demonstrate the potential return on investment in terms of improved patient outcomes and reduced inpatient ICU admission.^13^ To our knowledge, no published studies have quantified the impact of these ED-based ICU delivery models on patient outcomes and ICU admissions.

In 2015, the University of Michigan Health System created The Joyce and Don Massey Family Foundation Emergency Critical Care Center (EC3), an ICU within the ED of its flagship adult hospital. The objective of this study was to examine patient outcomes and resource use before and after implementation of this ED-based ICU model. We hypothesized that implementation of the EC3 would be associated with decreased mortality among the overall ED patient population and decreased inpatient ICU admissions from the ED.

## Methods

### Context, Interventions, and Study of the Interventions

This is a cohort analysis of patient outcomes and resource use before and after the implementation of an ED-based ICU at an academic medical center. The institutional review board at the University of Michigan reviewed and approved this study, which included a waiver of Health Insurance Portability and Accountability Act authorization. This study used retrospective data collected during the course of routine clinical care, and we treated all the data in a manner compliant with the Security Rule and the Privacy Rule of the Health Insurance Portability and Accountability Act. This study is reported in compliance with the Standards for Quality Improvement Reporting Excellence (SQUIRE) reporting guideline.^14^

The University of Michigan adult ED is part of a large academic medical center with approximately 75 000 adult ED visits per year. The EC3 ED-based ICU opened on February 16, 2015, as a 7800–square foot unit (to convert to meters squared, multiply by 0.09) consisting of 5 resuscitation or trauma bays and 9 patient rooms immediately adjacent to the main ED. The multidisciplinary EC3 team consisted of emergency medicine (EM) physicians (with or without critical care fellowship training), house staff (primarily EM residents and critical care fellows), physician assistants (with critical care training), ED nurses (with additional ICU training), respiratory therapists, and pharmacists. The unit is continuously staffed by 1 EM attending physician and 1 or 2 residents, fellows, or physician assistants. The patient-to-nurse ratio is 2:1. Evidence-based patient care pathways (eTable 1 in the Supplement) were designed to achieve timely delivery of ICU-level care in the ED setting and seamless transition to inpatient ICUs.

**Figure 1.**
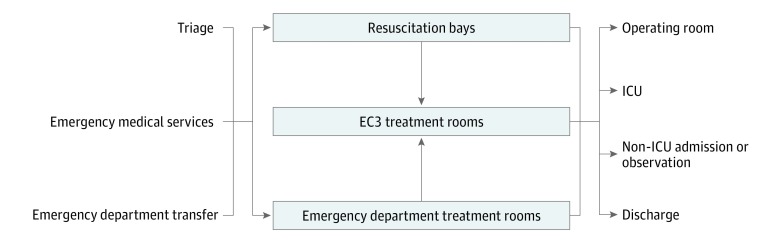
Emergency Critical Care Center (EC3) Patient Flow Diagram

All ED patients were initially evaluated and treated by the main ED team. Prior to EC3 implementation, all patients requiring ongoing critical care continued to be evaluated and treated by the main ED team in resuscitation bays or regular ED rooms in consultation with the inpatient ICU teams until an inpatient ICU bed became available or the patient no longer required critical care and was admitted to a non-ICU ward. After EC3 implementation, patients requiring ongoing critical care could be transferred to the EC3 team and cared for in the 9-bed ED-based ICU, even if inpatient ICU beds were available. Common indications for transfer to EC3 included severe sepsis or septic shock; altered mental status; overdose; major electrolyte disturbances, including diabetic ketoacidosis; gastrointestinal tract bleeding; respiratory distress or failure; congestive heart failure; and undifferentiated hypotension. Figure 1 illustrates how the EC3 is integrated into the overall flow of ED patients.

To test for differences in patient outcomes and resource use before and after EC3 implementation, we analyzed data from the electronic health records (EHRs) of all ED visits from September 2, 2012, through July 31, 2017. The pre-EC3 cohort was defined as all visits to the ED from September 2, 2012, through February 15, 2015 (897 days), and the post-EC3 cohort was defined as all visits to the ED from February 16, 2015, through July 31, 2017 (897 days). This period was selected because a new EHR system was deployed on September 2, 2012, and defining the pre-EC3 and post-EC3 cohorts in this fashion allowed for an identical number of days in each. All ED visits involving a documented encounter between a patient and an ED clinician that occurred during the pre-EC3 and post-EC3 periods were included in the analysis.

### Measures

The primary mortality outcome was 30-day mortality among all ED patients before and after EC3 implementation. We also examined 30-day mortality after hospital admission, in-hospital mortality, 24-hour mortality, and mortality prior to hospital admission. Mortality was determined based on death date or confirmed to be alive in the EHR. Patients with unknown mortality status in the EHR were assumed to be alive at 30 days.

The primary ICU admission outcome was ICU admission rate before and after EC3 implementation. Other ICU admission outcomes included short-stay ICU admissions and transfer to the ICU within 24 hours of admission to a non-ICU bed from the ED. Intensive care unit admission was defined as a patient physically occupying an inpatient ICU bed. To our knowledge, there is no agreement on the definition of *short-stay ICU admission*, so for the purposes of this analysis, we used the definition of any patient admitted from the ED to an inpatient ICU with a subsequent ICU LOS less than 24 hours.

### Statistical Analysis

We analyzed all visits from the entire ED population to prevent patient selection bias. Bivariate and multiple logistic regression analyses^15^ were used to test hypotheses about pre-EC3 and post-EC3 cohort differences in mortality and ICU admission outcomes. Covariates in the multivariable models included sex, age at ED visit (in 5-year increments), a modified version of the Charlson Comorbidity Index,^16^ and the Emergency Severity Index, a triage algorithm that assigns a score from 1, indicating the most urgent, through 5, indicating the least urgent.^17^ Receiver operating characteristic curves and the C statistic measuring the area under the receiver operating characteristic curve were used to evaluate the predictive power of each model. Model-based predicted marginal probabilities, defined as the average probability across the observed values of other covariates in the model, and average marginal effects (AMEs) of cohort, defined as the average difference between the 2 cohorts in the predicted probability of a given outcome,^18^ were calculated and converted to percentages to gain perspective on the magnitude of pre-EC3 and post-EC3 cohort differences. An α level of .05 was used for all analyses, and all hypothesis tests were 2-sided. Unadjusted and adjusted odds ratios (ORs) and 95% CIs were calculated, and the number needed to treat (NNT) was estimated based on adjusted risk differences.^19^ Cluster-robust SEs were estimated to account for multiple visits clustered within patients. Analyses were conducted with SAS statistical software version 9.4 (SAS Institute) and Stata statistical software version 15 (StataCorp). Data analyses were conducted from March 2, 2018, to May 28, 2019.

## Results

### Characteristics of Study Participants

A total of 349 310 patient encounters (mean [SD] age, 48.5 [19.7] years; 189 709 [54.3%] women) were identified and analyzed. There were 168 877 patients (92 038 [54.5%] women) in the pre-EC3 cohort and 180 433 patients (97 671 [54.1%] women) in the post-EC3 cohort (Table 1). Consistent with national trends, the mean (SD) volume of ED visits increased through time, from 188 (19.1) ED visits per day pre-EC3 to 201 (18.3) ED visits per day post-EC3, an increase of 6.8%. The mean (SD) daily census of the EC3 was 6.9 (2.1) patients treated by EC3 clinicians and 4.1 (3.2) patients as overflow from the main ED treated by the main ED clinicians. The EC3 occupancy by day of week and hour of day and mean daily census by month are reported in eTable 2 and eFigure 1 in the Supplement, respectively. Patients in the post-EC3 cohort had a mean age of 1 year older than those in the pre-EC3 cohort (mean [SD] age, 49.0 [19.7] vs 48.0 [19.7] years, respectively). The mean Charlson Comorbidity Index was 20.0% greater in the post-EC3 cohort compared with the pre-EC3 cohort (mean [SD], 2.4 [3.2] vs 2.0 [2.9]), reflective of increases in age and comorbidity through time. Increases in patient acuity (as indicated by lower Emergency Severity Index scores) and longer ED LOS were also observed in the post-EC3 cohort compared with the pre-EC3 cohort.

**Table 1.** Patient Demographic and ED Characteristics

Characteristic	No. (%)	
	Pre-EC3 Cohort	Post-EC3 Cohort
Total ED visits	168 877 (48.3)	180 433 (51.7)
ED visits, No./y	68 777	73 452
Patients treated in EC3	NA	6200 (3.4)
Age, mean (SD), y	48.0 (19.7)	49.0 (19.7)
Women	90 238 (54.5)	97 671 (54.1)
Triaged to ED resuscitation bay	11 429 (6.8)	14 251 (7.9)
Emergency Severity Index score, mean (SD)	2.65 (0.7)	2.61 (0.7)
Charlson Comorbidity Index score, mean (SD)	2.0 (2.9)	2.4 (3.2)
ED LOS, mean (SD), h	5.6 (4.0)	6.4 (4.4)
EC3 LOS, mean (SD), h	NA	9.4 (6.2)
ED admissions	62 838 (37.3)	69 431 (38.7)
Patients with missing 30-d mortality status	21 548 (12.8)	28 459 (15.8)
EC3 visits requiring mechanical ventilation	NA	1892 (30.5)
EC3 visits requiring vasopressors	NA	820 (13.2)

Abbreviations: EC3, Emergency Critical Care Center; ED, emergency department; LOS, length of stay; NA, not applicable.

The median (interquartile range) time to ICU-level care (EC3 or inpatient ICU) was 5.3 (3.4-7.9) hours in the pre-EC3 cohort and 3.4 (2.1-5.6) hours in the post-EC3 cohort, a difference of 1.9 hours (*t*_14,669_ = 25.6; *P* < .001). The number of patients who received ICU-level care within 6 hours of ED presentation increased abruptly with implementation of EC3 from 3142 patients (58.3%; 95% CI, 57.0%-59.7%) to 7205 patients (77.6%; 95% CI, 76.7%-78.4%) (χ^2^_1_ = 608.4; *P* < .001), a difference of 19.3 percentage points (eFigure 2 in the Supplement). The mean (SD) time in ED until transfer to EC3 was 3.7 (2.8) hours, and the mean (SD) EC3 LOS was 9.4 (6.2) hours (median [interquartile range], 7.9 [5.0-12.4] hours; range, 0-52.6 hours). The mean (SD) EC3 LOS for patients admitted to a non-ICU bed was 9.8 (5.9) hours. The most frequently used evidence-based patient care pathways included undifferentiated critical illness (2939 patients [47.4%]), sepsis (955 patients [15.4%]), shortness of breath or respiratory failure (722 patients [11.6%]), and gastrointestinal tract bleeding (496 patients [8.0%]) (eTable 1 in the Supplement).

### Mortality

**Figure 2.**
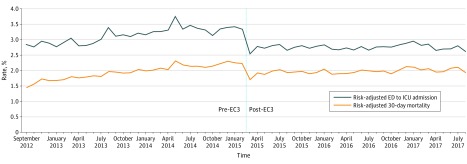
Risk-Adjusted 30-Day Mortality and Intensive Care Unit (ICU) Admission Rates Before and After Emergency Critical Care Center (EC3) Implementation ED indicates emergency department.

In the unadjusted analysis, there was no statistically significant bivariate association of cohort with 30-day mortality rate among all ED patients. However, results from the multivariable analysis in Table 2 indicated that, when other covariates were statistically controlled, the transition to EC3 was associated with a statistically significant 15.4% reduction in the odds of 30-day mortality (adjusted OR, 0.85; 95% CI, 0.80-0.90; *P* < .001; C = 0.86). The risk-adjusted 30-day mortality rate among all ED patients decreased from 2.13% pre-EC3 to 1.83% post-EC3 (NNT, 333 patient encounters; 95% CI, 256-476) (Table 2 and Figure 2), and the AME of cohort was −0.30% (95% CI, −0.41% to −0.19%; *P* < .001). As shown in Figure 2, the sharpest decline in risk-adjusted 30-day mortality rate occurred immediately after implementation of EC3. Restricting analysis to only patients with Emergency Severity Index scores of 1 or 2, implementation of EC3 was associated with a statistically significant reduction in the odds of 30-day mortality (adjusted OR, 0.87; 95% CI, 0.82-0.92; *P* < .001) (eTable 3 in the Supplement). In addition to the statistically significant association of EC3 implementation with reduced mortality, results indicated that older age (adjusted OR, 1.17; 95% CI, 1.16-1.18), male sex (adjusted OR, 1.16; 95% CI, 1.09-1.24), comorbidity (adjusted OR, 1.15; 95% CI, 1.16-1.17), and severity (adjusted OR, 5.32, 95% CI, 4.95-5.72) were independently associated with statistically significantly higher odds of 30-day mortality. The observed reduction in risk-adjusted 30-day mortality for 73 451 patients per year adult ED population equates to 220 lives saved per year after the implementation of EC3, or approximately 3 lives saved per 1000 ED visits (NNT, 333 patient encounters; 95% CI, 256-476).

**Table 2.** Pre-EC3 and Post-EC3 Mortality Rates

Outcome	Unadjusted			Risk-Adjusted^a^		
	Pre-EC3 Rate, %	Post-EC3 Rate, %	OR (95% CI)	Pre-EC3 Rate, %	Post-EC3 Rate, %	OR (95% CI)
30-d mortality						
All ED visits^b^	1.97	1.98	1.01 (0.95-1.06)	2.13	1.83	0.85 (0.80-0.90)
ED admissions^c^	4.52	4.39	0.97 (0.91-1.03)	4.74	4.18	0.87 (0.82-0.93)
ED admissions						
Hospital mortality^c^	2.16	2.07	0.96 (0.89-1.03)	2.20	2.00	0.90 (0.84-0.98)
24-h mortality^c^	0.30	0.22	0.74 (0.60-0.92)	0.29	0.22	0.74 (0.60-0.92)
Mortality prior to admission, all ED visits^b^	0.08	0.12	1.40 (1.13-1.74)	0.08	0.11	1.36 (1.09-1.71)

Abbreviations: EC3, Emergency Critical Care Center; ED, emergency department; OR, odds ratio.

^a^ Risk-adjusted percentages are from model-based predicted marginal probabilities, defined as the average probability of pre-EC3 and post-EC3 mortality across the observed values of other covariates in the model. Adjusted ORs are from multivariable analysis of the post-EC3 cohort as a predictor of mortality, statistically controlling for age, sex, Charlson Comorbidity Index score, and Emergency Severity Index score. Patients with missing data on the Emergency Severity Index (n = 370 [0.11%]) were excluded from multivariable analysis.

^b^ Total sample included 349 310 ED visits (pre-EC3, 168 877 ED visits; post-EC3, 180 433 ED visits) in which the patient was seen by an ED clinician.

^c^ Total sample included 132 269 ED visits (pre-EC3, 62 838 ED visits; post-EC3, 69 431 ED visits) in which the patient was seen by an ED clinician and subsequently admitted to the ED.

A similar pattern of lower mortality in the post-EC3 cohort among patients who were admitted to the hospital from the ED was observed in 30-day mortality, hospital mortality, and 24-hour mortality (Table 2; eFigure 3 in the Supplement). An exception to this pattern was observed in mortality prior to hospital admission, in which results showed that the post-EC3 cohort was associated with a small but statistically significant increase (adjusted OR, 1.36; 95% CI, 1.09-1.71) in the odds of mortality prior to hospital admission (Table 2; eFigure 3 in the Supplement).

### ICU Admissions

The unadjusted rate of ED admission to ICU per 100 000 ED visits decreased with implementation of EC3 by 12.9%, and results from the multivariable analysis in Table 3 indicated that, when other covariates were statistically controlled, the implementation of EC3 was associated with a statistically significant reduction in the odds of ED admission to ICU (adjusted OR, 0.80; 95% CI, 0.76-0.83; C = 0.84). The risk-adjusted rate of ED admission to ICU decreased from 3.2% in the pre-EC3 cohort to 2.7% in the post-EC3 cohort (Table 3; Figure 2) (NNT, 179; 95% CI, 149-217), and the AME of cohort was −0.56% (95% CI, −0.46% to −0.67%). As shown in Figure 2, consistent with the mortality results, the sharpest decline in risk-adjusted ED admission to ICU rate occurred immediately after implementation of EC3. In addition to the statistically significant difference of the post-EC3 cohort, we found that older age (adjusted OR, 1.07; 95% CI, 1.06-1.08), male sex (adjusted OR, 1.39; 95% CI, 1.33-1.46), comorbidity (adjusted OR, 0.98; 95% CI, 0.97-0.99), and severity (adjusted OR, 19.62; 95% CI, 18.50-20.81) were statistically significantly associated with odds of ED admission to ICU.

**Table 3.** Pre-EC3 and Post-EC3 ICU Use Rates

Outcome	Unadjusted			Risk-Adjusted^a^		
	Pre-EC3	Post-EC3	OR (95% CI)	Pre-EC3	Post-EC3	Adjusted OR (95% CI)
ED admissions to ICU per 100 000 ED visits, No. (%)^b^	3189 (3.2)	2778 (2.8)	0.87 (0.83-0.90)	3245 (3.2)	2684 (2.7)	0.80 (0.76-0.83)
Short-stay ICU admissions, %^c^	12.51	9.12	0.70 (0.62-0.80)	12.56	9.18	0.70 (0.62-0.80)
Transfer to ICU within 24 h of admission from ED to a non-ICU ward, %^d^	0.70	0.62	0.88 (0.77-1.02)	0.71	0.60	0.84 (0.73-0.97)

Abbreviations: EC3, Emergency Critical Care Center; ED, emergency department; ICU, intensive care unit; OR, odds ratio.

^a^ Risk-adjusted percentages are from model-based predicted marginal probabilities. Patients with missing data on the Emergency Severity Index (370 [0.11%]) were excluded from multivariable analysis. Adjusted ORs are from multivariable analysis of EC3 cohort as a predictor of mortality, statistically controlling for age, sex, Charlson Comorbidity Index score, and Emergency Severity Index score.

^b^ Total sample included 349 310 ED visits (pre-EC3, 168 877 ED visits; post-EC3, 180 433 ED visits) in which the patient was seen by a clinician.

^c^ Excludes ED admissions to procedural units (ie, operating room, interventional radiology, cardiac catheterization laboratory). Total sample included 10 398 ED visits (pre-EC3, 5386 ED visits; post-EC3, 5012 ED visits) in which the patient was seen by a clinician and subsequently admitted to the ICU.

^d^ Total sample included 121 871 ED visits (pre-EC3, 57 452 ED visits; post-EC3, 64 419 ED visits) in which the patient was seen by a clinician and subsequently admitted to a non-ICU ward.

As shown in Table 3, when other covariates were statistically controlled, the post-EC3 cohort was associated with a statistically significant reduction in the odds of short-stay ICU admission (ICU LOS <24 hours) (adjusted OR, 0.70; 95% CI, 0.62-0.80; C = 0.61), and the risk-adjusted short-stay ICU admission rate decreased from 12.6% in the pre-EC3 cohort to 9.2% in the post-EC3 cohort (AME, −3.4%; 95% CI, −4.6% to −2.2%). Older age (adjusted OR, 0.98; 95% CI, 0.96-0.99) and lower patient acuity (adjusted OR, 0.58; 95% CI, 0.52-0.65) were also associated with statistically significantly lower odds of short-stay ICU admission. In addition, the post-EC3 cohort was associated with a statistically significant reduction in the odds of transfer to ICU within 24 hours of admission to a non-ICU ward from the ED (adjusted OR, 0.84; 95% CI, 0.73-0.97; C = 0.65), and the risk-adjusted rate of transfer to ICU within 24 hours of admission to a non-ICU ward from the ED declined from 0.71% in the pre-EC3 to 0.60% in the post-EC3 (AME, −0.11%; 95% CI, −0.21% to −0.02%).

## Discussion

Results from this study at an academic medical center support our hypothesis that implementation of an ED-based ICU is associated with decreased mortality and decreased inpatient ICU admissions among ED patients. To our knowledge, this is the first report to demonstrate the potential benefits associated with this care delivery model.

### Mortality Reduction

Previous studies have demonstrated that patients who are critically ill and experience prolonged LOS (>6 hours) in the ED have increased mortality.^4,5,6,7,8,9,10^ The populations observed in these studies were heterogeneous in underlying disease processes, but the association of prolonged ED care with outcome was consistent. This association is not unexpected considering how the traditional US model of ED care is facing increasing demands for critical care delivery that can exceed resources, capacity, and capabilities. To address this challenge, we created an ED-based ICU care delivery model that provides dedicated space and resources combined with nurse and physician staffing based on traditional inpatient ICU care, designed to optimize time-sensitive diagnosis and treatment for patients who are critically ill or injured. Controlling for severity of illness, this strategy was associated with significant reductions in mortality and inpatient ICU admission. The variables potentially associated with improved survival are multifactorial and will require more in-depth analysis. However, the 1.9-hour reduction in median time to ICU-level care and 19.3% absolute increased proportion of patients receiving ICU-level care within 6 hours are potential factors.

Although more patients in the post-EC3 cohort died prior to admission, this may be associated with the significant number of patients receiving end-of-life care in the EC3 who would have otherwise been admitted to an inpatient bed. The EC3 offers the opportunity to thoroughly discuss goals of care and code status and to involve patients and treating physicians (eg, oncologists) in clinical decision-making. This often leads to initiation of comfort care measures while in EC3 rather than after admission to the ICU, which is another unique outcome benefit observed with implementation of EC3.

### ICU Resource Optimization

With implementation of EC3, many patients who would traditionally be admitted to an inpatient ICU were stabilized for non-ICU admission or, in some instances, discharged, decreasing the rate of inpatient ICU admission. The final disposition of patients treated in EC3 is presented in eFigure 4 in the Supplement. During the observed before and after periods, there was a clear demarcation in the inpatient ICU admission rate at the time EC3 opened. The unadjusted inpatient ICU admission rate was observed to decrease by 12.9%. This reduction occurred with contemporaneous increases in total ED volume, overall admission rate, patient acuity, and patient age. These findings indicate that the mean severity of illness of the patient population increased during the study; thus, the observed reduction of ICU admissions was not associated with a significant decrease in patient acuity.

The observed reduction in ICU admission rate is likely multifactorial, and a large factor in this reduction can potentially be explained by the association EC3 had with short-stay ICU admissions. We observed a 30% reduction in the odds of short-stay ICU admissions. Our initial pre-EC3 short-stay admission rate was relatively low at 12.5%, compared with reports as high as 40% of ICU admissions defined as short stays nationally.^20^ By avoiding short-stay admissions, the inpatient ICU can optimize bed resource allocation by providing capacity for patients who are decompensating in the wards or by increasing transfers from ICUs in other hospitals.

### Safety

To assess whether the observed decreased ICU admission rate was associated with an increased safety risk assumed by non-ICU wards, we analyzed emergent transfers from non-ICU wards to the ICU within the first 24 hours after admission from the ED. This did not change with implementation of EC3. Moreover, risk-adjusted 24-hour mortality of ED patients was observed to decrease with implementation of EC3. These 2 metrics are reassuring that this model of ED-based critical care can safely downgrade patients who are critically ill to non-ICU wards without exposing them to added risk of early decompensation or death.

### Significance

In 1961, Safar et al^21^ ushered in the era of inpatient critical care by describing the first US inpatient ICU at Baltimore City Hospital. In the same year, the first group of physicians focused their practice on EM in Alexandria, Virginia.^22^ Since then, critical care and EM have evolved as separate specialties. In the last 2 decades, both specialties have faced the increased challenge of providing timely and effective care for patients who are the most critically ill or most severely injured.^3^ However, providing prolonged critical care in the traditional ED environment is challenging and associated with increased mortality.^4,5,6,7,8,9,10,23^ In this study, we describe a novel health care delivery strategy that provides contemporary evidence-based critical care and ICU resources within the ED. This *Right Care, Right Now* approach is championed by the Society of Critical Care Medicine.^24^ In fact, one of the founders of critical care, Ake Grenvik, MD, PhD, predicted decades ago that “Many critically ill patients no longer need admission to the hospital if the diagnostic work-up and treatment may be completed in an ED short-term ICU.”^25^ While other strategies for delivering critical care to ED patients are being explored,^11,12^ to our knowledge, this is the first report in which an alternative critical care model has been associated with improved survival and resource use. Additional studies are needed to determine the effect of this model in other health care systems.

### Limitations

A major limitation of this study is the pragmatic nature of its design. It is a single-center, uncontrolled, before-and-after design caring for a heterogeneous population in both disease and operational complexity. There are potential covariates that could contribute to the differences observed in the pre-EC3 and post-EC3 results. We attempted to control for as many of these as possible; however, there may have been other variables that were unaccounted for that contributed to the observed results. There is no accepted standard for risk adjustment of patients who are critically ill within the ED setting. Traditional scoring systems have been validated in ICU cohorts only and are poor predictors of mortality when used in the ED. We used an adjusted Charlson Comorbidity Index as described by Quan et al^16^ to address the complexity and comorbid illness of our patient population. To increase the strength of the model, we also included age and the Emergency Severity Index score.^17^ Although not validated for mortality prediction, this measure identifies a representative range of acuity in the ED patient population. The 30-day mortality analysis was potentially limited by the assumption that patients with unknown mortality status were alive 30 days after ED presentation. However, when analysis was performed excluding the patients with unknown mortality status at 30 days, the differences observed between risk-adjusted 30-day mortality of all ED patients and returning ED patients remained similar in magnitude and statistically significant (eTable 4 in the Supplement). Statistically significant differences observed in this study could partially be attributable to large sample sizes and should be interpreted appropriately. An incremental rollout of a new EHR system occurred between 2012 and 2014, which may have affected risk-adjusted mortality in the pre-EC3 period. We cannot rule out the possibility that simply increasing the number of clinicians providing care in the post-EC3 period contributed to improved survival. Additionally, it is possible that secular trends in disease-specific mortality could have contributed to outcome differences in the pre-EC3 and post-EC3 cohorts.

## Conclusions

Implementation of a novel ED-based ICU was associated with decreased risk-adjusted mortality and inpatient ICU admission rates among ED patients. Additional research is warranted to determine the value of this novel care delivery model in other health care systems. If translatable to other systems, this model may be an effective strategy to address the challenge of increasing demand for critical care delivery that is exceeding ICU capacity in the US health care system.

## Notes

Supplement. **eTable 1.** Emergency Critical Care Center Disease-Specific Pathways **eTable 2.** Emergency Critical Care Center Occupancy by Hour of Day and Day of Week **eTable 3.** Pre-EC3 and Post-EC3 Mortality Rates From Bivariate and Multivariable Analysis, Restricted to Emergency Severity Index Scores of 1 or 2 **eTable 4.** Pre-EC3 and Post-EC3 Mortality Rates From Bivariate and Multivariable Analysis Excluding Patients With Unknown Status **eFigure 1.** Emergency Critical Care Center (EC3) Mean Daily Census by Month **eFigure 2.** Emergency Department (ED) Visits Receiving Intensive Care Unit (ICU)-Level Care Within 6 Hours of ED Arrival **eFigure 3.** Monthly Mortality Metrics Before and After Emergency Critical Care Center (EC3) Implementation **eFigure 4.** Disposition of Emergency Critical Care Center (EC3) Visits

## References

[1] Barrett ML, Smith MW, Elixhauser A, Honigman LS, Pines JM Utilization of intensive care services, 2011 In: Healthcare Cost and Utilization Project (HCUP) Statistical Briefs. Rockville, MD: Agency for Healthcare Research and Quality; 2006. 25654157

[2] Halpern SD ICU capacity strain and the quality and allocation of critical care. Curr Opin Crit Care. 2011;17(6):-. doi:10.1097/MCC.0b013e32834c7a53 21986461

[3] Herring AA, Ginde AA, Fahimi J, Increasing critical care admissions from US emergency departments, 2001-2009. Crit Care Med. 2013;41(5):1197-1204. doi:10.1097/CCM.0b013e31827c086f 23591207 PMC3756824

[4] Chalfin DB, Trzeciak S, Likourezos A, Baumann BM, Dellinger RP; DELAY-ED study group Impact of delayed transfer of critically ill patients from the emergency department to the intensive care unit. Crit Care Med. 2007;35(6):1477-1483. doi:10.1097/01.CCM.0000266585.74905.5A 17440421

[5] Singer AJ, Thode HC Jr, Viccellio P, Pines JM The association between length of emergency department boarding and mortality. Acad Emerg Med. 2011;18(12):1324-1329. doi:10.1111/j.1553-2712.2011.01236.x 22168198

[6] Rincon F, Mayer SA, Rivolta J, Impact of delayed transfer of critically ill stroke patients from the emergency department to the neuro-ICU. Neurocrit Care. 2010;13(1):75-81. doi:10.1007/s12028-010-9347-0 20428969

[7] Bhat R, Goyal M, Graf S, Impact of post-intubation interventions on mortality in patients boarding in the emergency department. West J Emerg Med. 2014;15(6):708-711. doi:10.5811/westjem.2014.7.22292 25247049 PMC4162735

[8] Carr BG, Kaye AJ, Wiebe DJ, Gracias VH, Schwab CW, Reilly PM Emergency department length of stay: a major risk factor for pneumonia in intubated blunt trauma patients. J Trauma. 2007;63(1):9-12. doi:10.1097/TA.0b013e31805d8f6b 17622862

[9] Hung SC, Kung CT, Hung CW, Determining delayed admission to intensive care unit for mechanically ventilated patients in the emergency department. Crit Care. 2014;18(4):485. doi:10.1186/s13054-014-0485-1 25148726 PMC4175615

[10] Cha WC, Cho JS, Shin SD, Lee EJ, Ro YS The impact of prolonged boarding of successfully resuscitated out-of-hospital cardiac arrest patients on survival-to-discharge rates. Resuscitation. 2015;90(90):25-29. doi:10.1016/j.resuscitation.2015.02.004 25680821

[11] Scalea TM, Rubinson L, Tran Q, Critical care resuscitation unit: an innovative solution to expedite transfer of patients with time-sensitive critical illness. J Am Coll Surg. 2016;222(4):614-621. doi:10.1016/j.jamcollsurg.2015.12.060 26920992

[12] Weingart SD, Sherwin RL, Emlet LL, Tawil I, Mayglothling J, Rittenberger JC ED intensivists and ED intensive care units. Am J Emerg Med. 2013;31(3):617-620. doi:10.1016/j.ajem.2012.10.015 23380127

[13] Gunnerson KJ The emergency department’s impact on inpatient critical care resources. Acad Emerg Med. 2017;24(10):1283-1285. doi:10.1111/acem.13268 28772343

[14] Ogrinc G, Davies L, Goodman D, Batalden P, Davidoff F, Stevens D. SQUIRE 2.0 (Standards for QUality Improvement Reporting Excellence): revised publication guidelines from a detailed consensus process. BMJ Qual Saf. 2016;25(12):986-992. doi:10.1136/bmjqs-2015-004411 26369893 PMC5256233

[15] Agresti A Categorical Data Analysis. 3rd ed Hoboken, NJ: John Wiley & Sons; 2013.

[16] Quan H, Li B, Couris CM, Updating and validating the Charlson comorbidity index and score for risk adjustment in hospital discharge abstracts using data from 6 countries. Am J Epidemiol. 2011;173(6):676-682. doi:10.1093/aje/kwq433 21330339

[17] Gilboy N, Tanabe T, Travers D, Rosenau AM Emergency Severity Index (ESI): A Triage Tool For Emergency Department Care, Version 4. Rockville, MD: Agency for Healthcare Research and Quality; 2011.

[18] Norton EC, Dowd BE, Maciejewski ML Marginal effects: quantifying the effect of changes in risk factors in logistic regression models. JAMA. 2019;321(13):1304-1305. doi:10.1001/jama.2019.1954 30848814

[19] Bender R, Kuss O, Hildebrandt M, Gehrmann U Estimating adjusted NNT measures in logistic regression analysis. Stat Med. 2007;26(30):5586-5595. doi:10.1002/sim.3061 17879268

[20] Chidi OO, Perman SM, Ginde AA Characteristics of short-stay critical care admissions from emergency departments in Maryland. Acad Emerg Med. 2017;24(10):1204-1211. doi:10.1111/acem.13188 28323374 PMC5967876

[21] Safar P, Dekornfeld TJ, Pearson JW, Redding JS The intensive care unit: a three year experience at Baltimore city hospitals. Anaesthesia. 1961;16:275-284. doi:10.1111/j.1365-2044.1961.tb13827.x 13745344

[22] Zink BJ Anyone, Anything, Anytime: A History of Emergency Medicine. 2nd ed Dallas, TX: American College of Emergency Physicians; 2018.

[23] Halpern NA, Pastores SM Critical care medicine in the United States 2000-2005: an analysis of bed numbers, occupancy rates, payer mix, and costs. Crit Care Med. 2010;38(1):65-71. doi:10.1097/CCM.0b013e3181b090d0 19730257

[24] Angood PB Right care, right now—you can make a difference. Crit Care Med. 2005;33(12):2729-2732. doi:10.1097/01.CCM.0000194537.13327.4B 16352951

[25] Grenvik A, Ayres SM, Holbrook PR, Shoemaker WC, eds. Textbook of Critical Care. 4th ed Philadelphia, PA: WB Saunders; 2000.

