# Supplementary material for: Association of an Emergency Department–Based Intensive Care Unit With Survival and Inpatient Intensive Care Unit Admissions
Source: JAMA Netw Open. 2019 Jul 24;2(7):e197584. doi: 10.1001/jamanetworkopen.2019.7584 (PMC6659143; doi:10.1001/jamanetworkopen.2019.7584)
Supplement: Supplement. — eTable 1. Emergency Critical Care Center Disease-Specific Pathways eTable 2. Emergency Critical Care Center Occupancy by Hour of Day and Day of Week eTable 3. Pre-EC3 and Post-EC3 Mortality Rates From Bivariate and Multivariable Analysis, Restricted to Emergency Severity Index Scores of 1 or 2 eTable 4. Pre-EC3 and Post-EC3 Mortality Rates From Bivariate and Multivariable Analysis Excluding Patients With Unknown Status eFigure 1. Emergency Critical Care Center (EC3) Mean Daily Census by Month eFigure 2. Emergency Department (ED) Visits Receiving Intensive Care Unit (ICU)-Level Care Within 6 Hours of ED Arrival eFigure 3. Monthly Mortality Metrics Before and After Emergency Critical Care Center (EC3) Implementation eFigure 4. Disposition of Emergency Critical Care Center (EC3) Visits [file jamanetwopen-2-e197584-s001.pdf]

Supplementary Online Content

Gunnerson KJ, Bassin BS, Havey RA, et al. Association of an emergency department–based intensive care unit with survival and inpatient intensive care unit admissions. *JAMA Netw Open*. 2019;2(7):e197584. doi:10.1001/jamanetworkopen.2019.7584

eTable 1. Emergency Critical Care Center Disease-Specific Pathways

eTable 2. Emergency Critical Care Center Occupancy by Hour of Day and Day of Week

eTable 3. Pre-EC3 and Post-EC3 Mortality Rates From Bivariate and Multivariable Analysis, Restricted to Emergency Severity Index Scores of 1 or 2

eTable 4. Pre-EC3 and Post-EC3 Mortality Rates From Bivariate and Multivariable Analysis Excluding Patients With Unknown Status

eFigure 1. Emergency Critical Care Center (EC3) Mean Daily Census by Month

eFigure 2. Emergency Department (ED) Visits Receiving Intensive Care Unit (ICU)-Level Care Within 6 Hours of ED Arrival

eFigure 3. Monthly Mortality Metrics Before and After Emergency Critical Care Center (EC3) Implementation

eFigure 4. Disposition of Emergency Critical Care Center (EC3) Visits

This supplementary material has been provided by the authors to give readers additional information about their work.

© 2019 Gunnerson KJ et al. *JAMA Network Open.*

eTable 1. Emergency Critical Care Center Disease-Specific Pathways

**EC3 Pathway** *Multiple per patient possible*	**Count**	**%**
BiPap/Intubation/Vent	672	10.8%
Diabetic Ketoacidosis	350	5.6%
End of Life	89	1.4%
Gastrointestinal Bleed	496	8.0%
Minor Intracranial Hemorrhage	10	0.2%
Post Cardiac Arrest	106	1.7%
Sepsis	955	15.4%
Shortness of Breath	722	11.6%
Status Epilepticus	70	1.1%
Subarachnoid Hemorrhage	165	2.7%
Undifferentiated	2,939	47.4%
No Pathway Selected	465	7.5%

EC3: Emergency Critical Care Center

© 2019 Gunnerson KJ et al. *JAMA Network Open.*

eTable 2. Emergency Critical Care Center Occupancy by Hour of Day and Day of Week

EC3 occupancy by hour, broken down by hour of day and day of week for the 85th percentile of occupancy in the EC3 beds.

	0:00	1:00	2:00	3:00	4:00	5:00	6:00	7:00	8:00	9:00	10:00	11:00	12:00	13:00	14:00	15:00	16:00	17:00	18:00	19:00	20:00	21:00	22:00	23:00
Sunday	4	4	5	4	4	3	3	3	4	3	3	3	4	4	4	4	3	4	4	3	3	3	3	3
Monday	3	3	4	4	4	4	4	4	5	4	4	5	6	6	6	6	7	6	6	7	6	6	7	6
Tuesday	6	7	7	8	7	6	6	6	6	6	6	6	6	6	6	6	6	5	7	7	6	6	6	6
Wednesday	7	7	7	8	8	7	7	7	6	6	6	6	7	7	7	6	6	7	7	7	7	6	5	5
Thursday	5	6	6	7	7	6	5	5	5	6	6	5	6	5	5	6	6	6	6	7	8	7	7	7
Friday	6	7	6	5	6	6	6	5	5	6	5	6	6	7	7	6	6	7	7	6	6	6	5	5
Saturday	6	6	6	6	5	4	4	4	5	5	5	5	4	4	5	5	6	6	5	5	4	4	5	4

The Michigan Medicine Emergency Department uses the 85th percentile of bed occupancy to determine appropriate nurse staffing. The dates between 2/15/2015 and 5/1/2015 are excluded from data for this graph because ongoing construction in EC3 resulted in only 5 of 9 beds being available during that time.

Beds Occupied in EC3
(Out of 9 available)

1 9

© 2019 Gunnerson KJ et al. *JAMA Network Open.*

eTable 3. Pre-EC3 and Post-EC3 Mortality Rates From Bivariate and Multivariable Analysis, Restricted to Emergency Severity Index Scores of 1 or 2

	Unadjusted				Risk-Adjusted[a]		
**Mortality Outcomes**	**Pre-EC3**	**Post-EC3**	**OR (95% CI)**		**Pre-EC3**	**Post-EC3**	**AOR (95% CI)**
30-day mortality, all ED visits[b] (%)	3.61	3.51	0.97 (0.92-1.03)		3.81	3.35	0.87 (0.82-0.93)
30-day mortality, ED admissions[c] (%)	5.76	5.56	0.96 (0.91-1.03)		6.00	5.37	0.89 (0.83-0.95)
Hospital mortality, ED admissions[c] (%)	2.92	2.75	0.94 (0.87-1.02)		2.97	2.70	0.91 (0.84-0.98)
24-hour mortality, ED admissions[c] (%)	0.40	0.24	0.59 (0.47-0.75)		0.39	0.24	0.61 (0.48-0.78)
Mortality prior to admission, all ED visits[b] (%)	0.17	0.23	1.34 (1.07-1.67)		0.17	0.23	1.35 (1.08-1.68)

[a]Risk-adjusted percentages are from model-based predicted marginal probabilities, defined as the average probability of pre- and post-EC3 mortality across the observed values of other covariates in the model.
[b]Sample size = 164,239 ED visits (76,604 Pre-EC3 and 87,635 Post-EC3) where patient was seen by an ED provider and ESI score = 1 or 2.
[c]Sample size = 89,915 ED visits (41,939 Pre-EC3 and 47,976 Post-EC3) where patient was seen by an ED provider and subsequently admitted and ESI score = 1 or 2.

Legend: OR = odds ratio for bivariate logistic regression analysis of EC3 cohort as a predictor of mortality. AOR = adjusted odds ratio from multivariable logistic regression analysis of EC3 cohort as a predictor of mortality, statistically controlling for age, sex, and Charlson Comorbidity Index. CI = confidence interval.

© 2019 Gunnerson KJ et al. *JAMA Network Open.*

eTable 4. Pre-EC3 and Post-EC3 Mortality Rates From Bivariate and Multivariable Analysis Excluding Patients With Unknown Status

	**Unadjusted**				**Risk-Adjusted[a]**		
	**Pre-EC3**	**Post-EC3**	**OR (95% CI)**		**Pre-EC3**	**Post-EC3**	**AOR (95% CI)**
30-day mortality, all ED patients[b] (%)	2.24	2.34	1.04 (0.96-1.05)		2.43	2.15	0.88 (0.83-0.92)
30-day mortality, ED admissions[c] (%)	4.92	4.89	0.99 (0.94-1.05)		5.14	4.66	0.90 (0.85-0.95)

[a]Risk-adjusted percentages are from model-based predicted marginal probabilities, defined as the average probability of pre- and post-EC3 mortality across the observed values of other covariates in the model. Cases with missing data on the Emergency Severity Index (*n*=318, 0.11%) were excluded from multivariable analysis.
[b]Sample size = 299,303 ED visits where patient was seen by a provider and 30-day mortality status was confirmed.
[c]Sample size = 119,047 ED visits where patient was seen by a provider, was subsequently admitted, and 30-day mortality status was confirmed.

Legend:
EC3: Emergency Critical Care Center
ED: Emergency Department
OR: odds ratio for bivariate analysis of EC3 cohort as a predictor of mortality
AOR: adjusted odds ratio from multivariable analysis of EC3 cohort as a predictor of mortality, statistically controlling for age, sex, Charlson Comorbidity Index, and Emergency Severity Index score
CI = confidence interval.

© 2019 Gunnerson KJ et al. *JAMA Network Open.*

eFigure 1. Emergency Critical Care Center (EC3) Mean Daily Census by Month

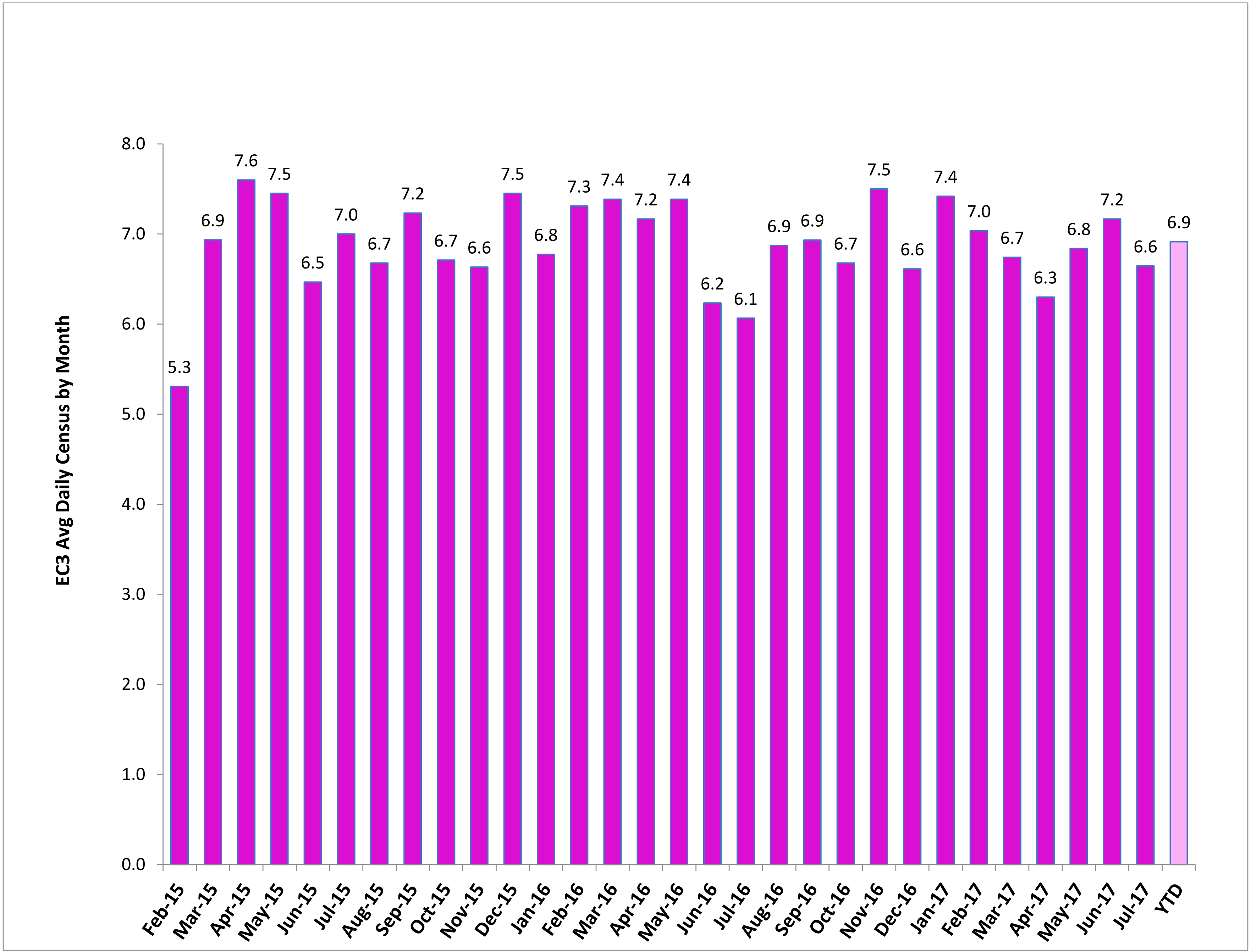

© 2019 Gunnerson KJ et al. *JAMA Network Open.*

eFigure 2. Emergency Department (ED) Visits Receiving Intensive Care Unit (ICU)-Level Care Within 6 Hours of ED Arrival

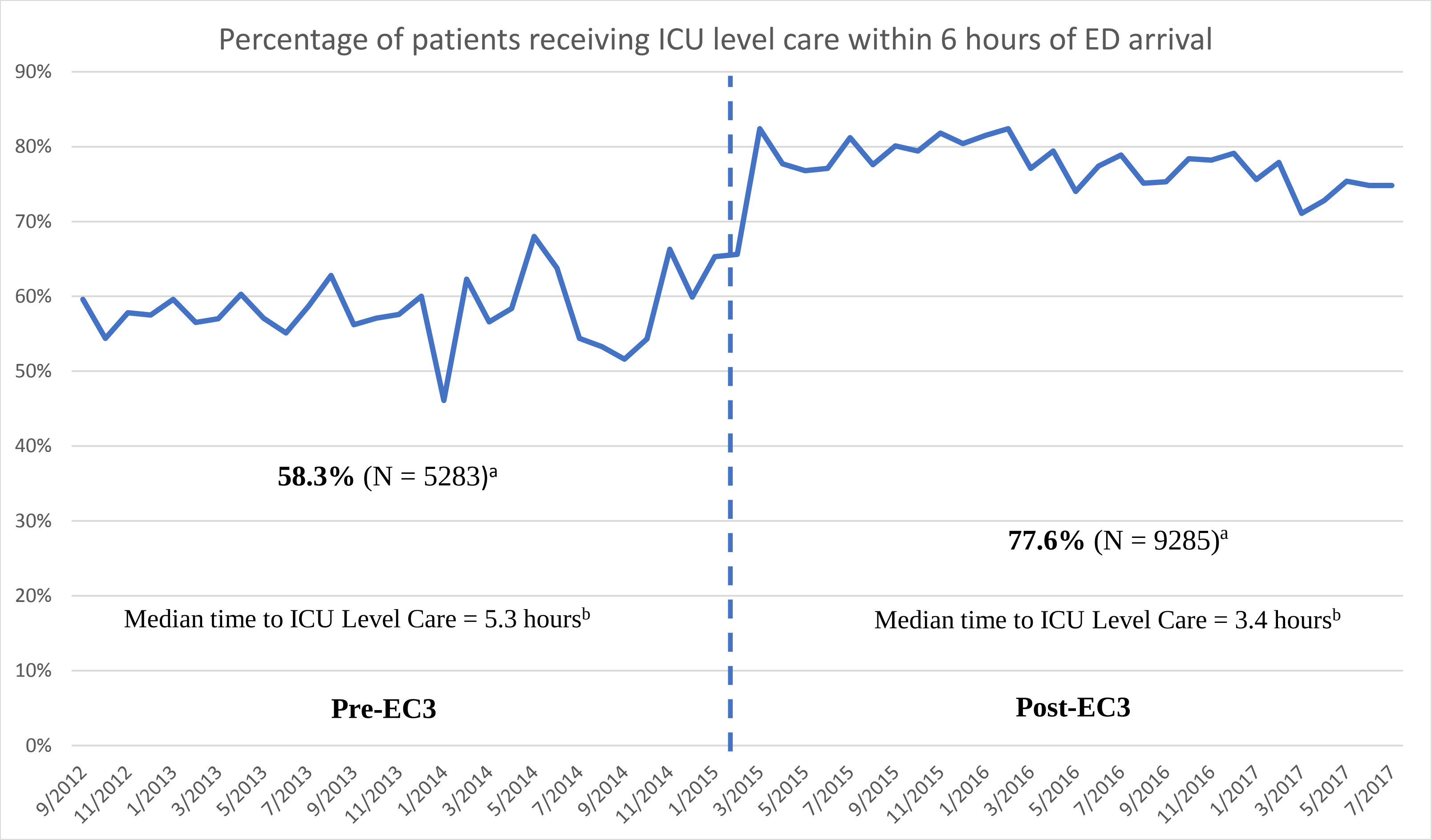

[a]Proportion of ED visits receiving ICU care within 6 hours of ED arrival, Pre-EC3 58.3% (95% CI 57.0-59.7) vs. Post-EC3 77.6% (95% CI 76.7-78.4), $\chi^2(1) = 608.4$, $p < .001$

[b]Median time from ED arrival to ICU-level care, Pre-EC3 5.3 hours (IQR=4.6) vs. Post-EC3 3.4 hours (IQR=3.5), $p < .001$

© 2019 Gunnerson KJ et al. *JAMA Network Open.*

eFigure 3. Monthly Mortality Metrics Before and After Emergency Critical Care Center (EC3) Implementation

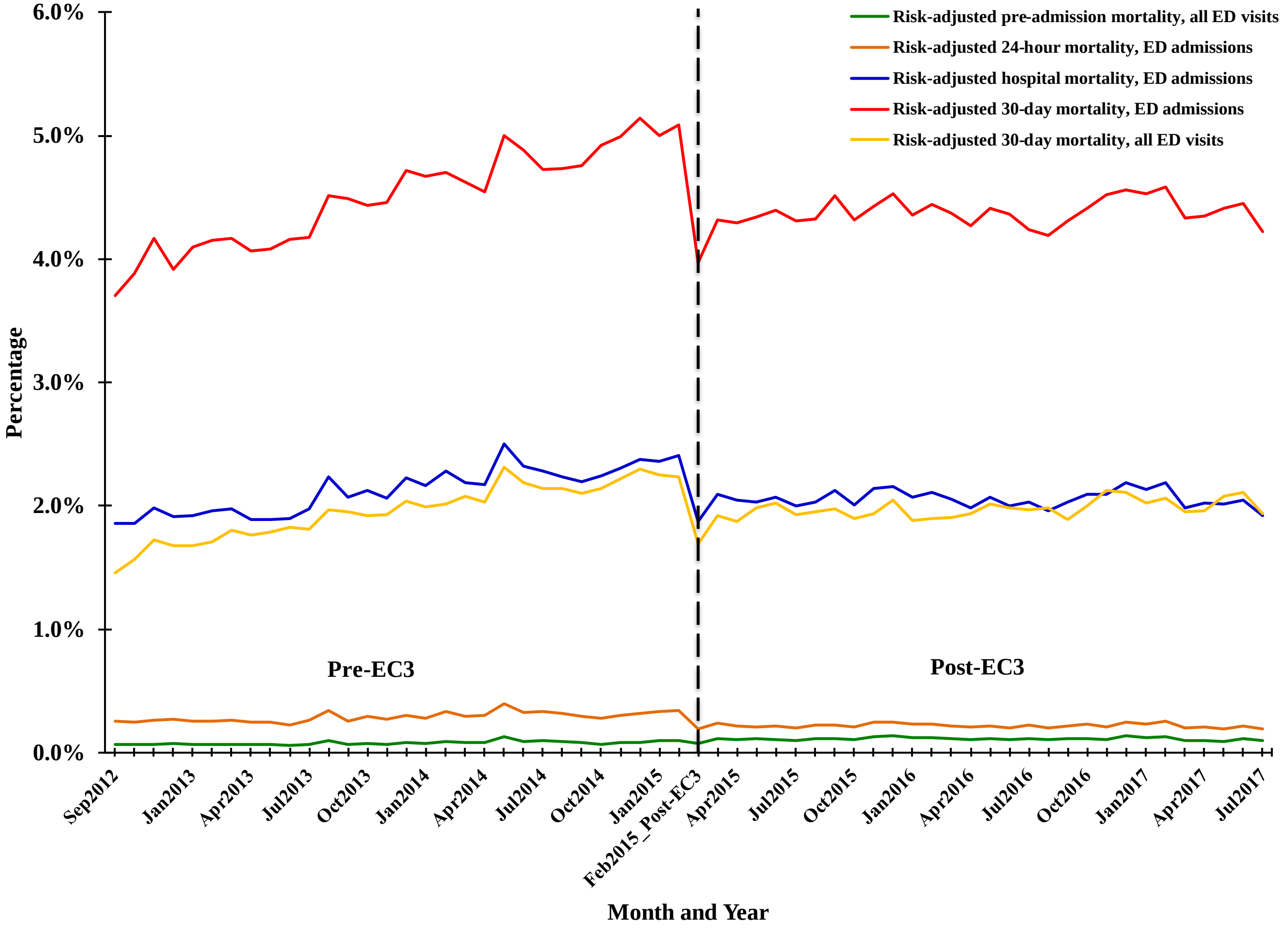

Legend:
EC3: Emergency Critical Care Center
ED: Emergency Department

© 2019 Gunnerson KJ et al. *JAMA Network Open.*

eFigure 4. Disposition of Emergency Critical Care Center (EC3) Visits

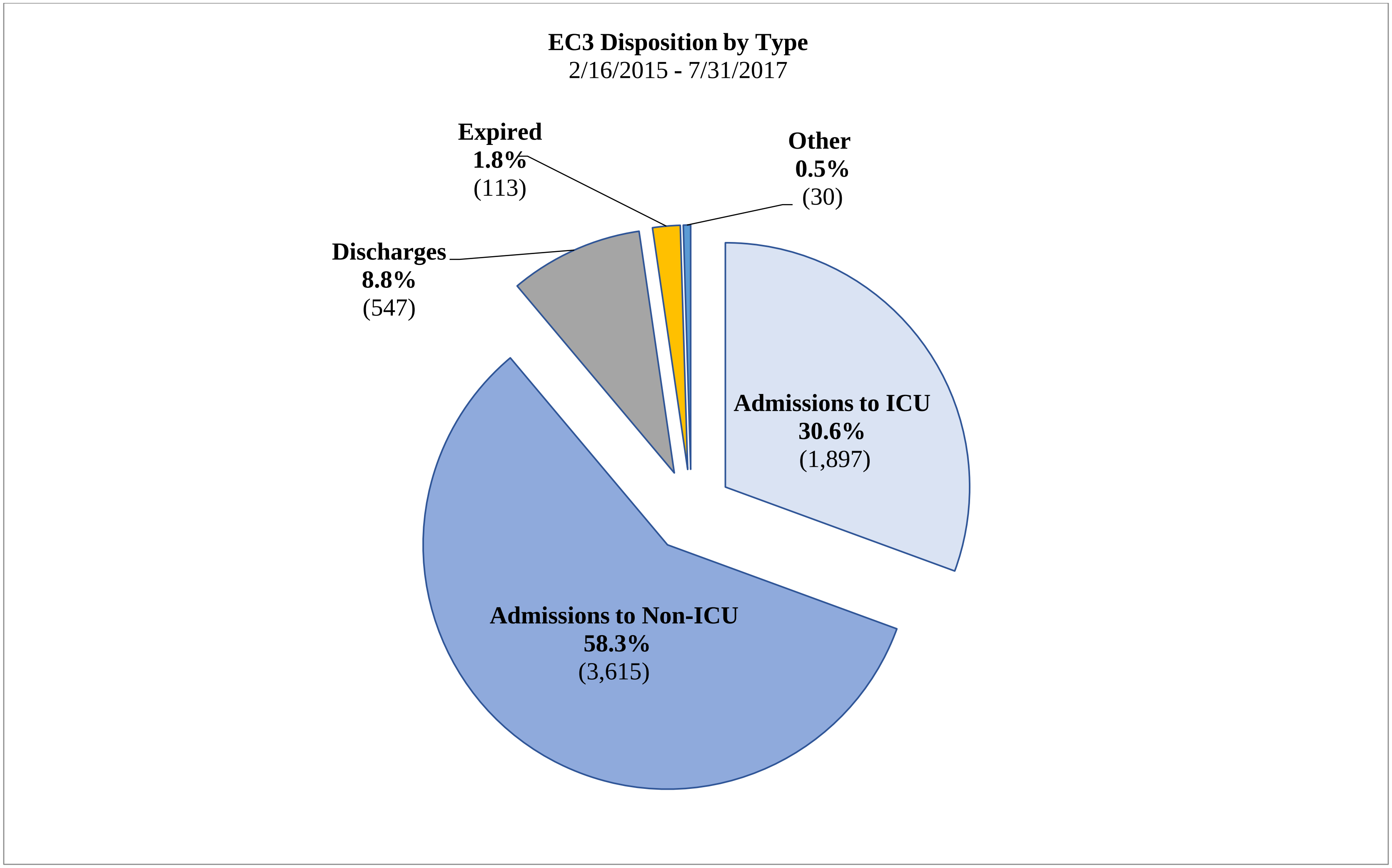

© 2019 Gunnerson KJ et al. *JAMA Network Open.*
